# Nurses’ workload during the COVID-19 pandemic: potential for experiences of moral distress

**DOI:** 10.1590/0034-7167-2023-0200

**Published:** 2024-03-18

**Authors:** Thallison Carlos Campos Santos, Gabriela da Costa Soares, Kelly Cristina Oliveira de Lima, Beatriz Bolognani Cardoso de Souza, Isabela Silva Câncio Velloso, Carolina da Silva Caram

**Affiliations:** I Universidade Federal de Minas Gerais. Belo Horizonte, Minas Gerais, Brazil

**Keywords:** Nurse, Ethics, Stress Disorders, Work, COVID-19., Enfermeras, Ética, Daño Moral, Trabajo, COVID-19., Enfermeiro, Ética, Dano Moral, Trabalho, COVID-19.

## Abstract

**Objectives:**

to understand nurses’ experiences of moral distress related to work overload during the COVID-19 pandemic in Brazil.

**Methods:**

qualitative research, whose data collection occurred through individual interviews with 19 nurses who worked on the front line of COVID-19 in health services in southeastern Brazil. Data were analyzed using thematic content analysis.

**Results:**

work overload proved to be a powerful source of experiences of moral distress due to excessive working hours during vaccination, double working hours, a troubled relationship due to pressure from managers and the population and physical and mental exhaustion, which prevented nurses from act according to their judgment.

**Final Considerations:**

nurses’ work overload reflects on quality patient care and prevents nurses from acting in accordance with their moral principles, generating moral distress in nurses.

## RESUMO

**Objetivos:**

compreender vivências de sofrimento moral de enfermeiros relacionadas à sobrecarga de trabalho durante a pandemia de COVID-19 no Brasil.

**Métodos:**

pesquisa qualitativa, cuja coleta de dados ocorreu através de entrevistas individuais com 19 enfermeiros que atuaram na linha de frente da COVID-19 em serviços de saúde da região Sudeste do Brasil. Os dados foram analisados mediante análise temática de conteúdo.

**Resultados:**

a sobrecarga de trabalho mostrou-se potente fonte para vivências de sofrimento moral devido à jornada de trabalho excessiva na vacinação, à dupla jornada de trabalho, à relação conturbada por pressão de gestores e população e ao esgotamento físico e mental, os quais impediam o enfermeiro de agir conforme o seu julgamento.

**Considerações Finais:**

a sobrecarga de trabalho dos enfermeiros reflete na assistência de qualidade ao paciente, e impede que os enfermeiros atuem conforme os seus princípios morais, gerando sofrimento moral nos enfermeiros.

## RESUMEN

**Objetivos:**

comprender las experiencias de angustia moral de los enfermeros relacionadas con la sobrecarga de trabajo durante la pandemia de COVID-19 en Brasil.

**Métodos:**

investigación cualitativa, cuya recolección de datos ocurrió a través de entrevistas individuales con 19 enfermeros que actuaron en la primera línea de COVID-19 en servicios de salud de la región Sudeste de Brasil. Los datos fueron analizados mediante análisis de contenido temático.

**Resultados:**

la sobrecarga de trabajo resultó ser una poderosa fuente de experiencias de sufrimiento moral por exceso de jornada durante la vacunación, doble jornada laboral, relaciones conflictivas por presiones de los directivos y la población y agotamiento físico y mental, lo que impidió al enfermero actuar según su criterio.

**Consideraciones Finales:**

la sobrecarga de trabajo de las enfermeras se refleja en la calidad de la atención al paciente e impide que las enfermeras actúen de acuerdo con sus principios morales, generando sufrimiento moral en las enfermeras.

## INTRODUCTION

The COVID-19 pandemic, decreed on March 11, 2020 and ended in May 2023 by the World Health Organization, was a global challenge for public health. The severity of the symptoms with which the disease occurred as well as its rapid transmission capacity were addressed as sources of overload on the health system infrastructure in most countries where the virus spread^(1)^.

In this context, the role of nurses in the face of the risks and challenges of a new disease stood out, having a strategic role at the institutional level with regard to action planning, physical structure optimization, human resources management, team training and construction of care flows and protocols^(2)^. It is also worth highlighting the care role of nurses on the front line of COVID-19, in a context of labor instability, with overload and precarious working conditions, with professionals being susceptible to risks of different natures^(3)^.

Among the risks and challenges that generate overloads that nurses had to experience during the pandemic, those related to the work environment’s organizational aspects stand out. Situations such as insufficient working conditions, lack of personnel and materials, work volume and dissatisfaction with work were cited in the literature^(4-6)^. It is noteworthy that patient care practice can be inappropriate due to the overload of work activities, compromising the quality of care and increasing the risk of moral distress (MD) for professionals^(7)^.

With regard to work overload, a survey that analyzed health professionals’ working conditions during the COVID-19 pandemic in Brazil identified that almost 50% of these professionals were subjected to work overload and the professionals who worked in direct care to infected patients had significant consequences in relation to mental health^(8)^. Another study that assessed nursing professionals’ living and working conditions facing the COVID-19 pandemic in Brazil found that the category presented unfavorable working conditions, mainly evidenced by work overload, low pay and compromised physical and mental health^(9)^.

The need to adapt to the reality of the pandemic scenario as well as the risks and challenges generated by increased demand in health service provision had short and medium-term consequences on health professionals’ mental health, especially nurses^(9-10)^. Although the effects of the pandemic on these professionals’ health are still under assessment, the risks and challenges to which they were exposed constitute moral problems and are potential causes of MD. MD can be understood as a painful feeling experienced by professionals when prevented from acting according to their moral conscience^(11)^, which arises on occasions in which they judge how they should act ethically and morally, but do not complete the action according to their discernment due to internal or external circumstances^(12)^.

MD is a unique and individual process that permeates nurses’ sensitivity and moral deliberation^(13)^. Considering its procedural nature, its consequences go beyond the painful feeling experienced at the time of the phenomenon, and can trigger fragility in the moral integrity of professionals, invisibilities, affect nurses’ mental health, among others^(5,14)^. Such consequences are related to the psychosocial work environment dimension, i.e., values and beliefs that interfere with professional practice^(14)^.

Faced with the extreme situations experienced, such as working in atypical conditions, marked by an increase in the number of patients, scarce resources and a high number of professional leaves of absence, nurses often assumed responsibility for the moral failures that occurred due to the adversities of the moment, even that these failures were inevitable and were beyond individuals’ desire^(15)^. Faced with the impossibility of acting in such situations, even carrying out judgment and outlining the best way to act, nurses ended up experiencing MD.

In order to understand existing scientific production in the databases, a search was carried out using the descriptors (Nursing) AND (Stress Disorders, Post-Traumatic) AND (COVID-19), and after reading the titles and abstracts, nine articles were selected. After complete reading, four address nursing work during the pandemic with factors that influenced the experience of MD among nurses, citing work overload and multiple shifts as possible generators of MD^(14,16-19)^.

Considering the above, the guiding question of this study arises: what are the experiences of MD among nurses related to work overload during the COVID-19 pandemic? Understanding the relationship between work overload and its consequences in nurses’ practice as a potential for MD experiences in health services during the COVID-19 pandemic can provide important support for rethinking the work environment organization, providing strategies for coping with MD.

## OBJECTIVES

To understand nurses’ experiences of MD related to work overload during the COVID-19 pandemic in Brazil.

## METHODS

### Ethical aspects

This research was submitted to the Research Ethics Committee the *Universidade Federal de Minas Gerais*, in accordance with the precepts of Resolution 466/12 of the Brazilian National Health Council. The study participants signed the Informed Consent Form (ICF) voluntarily after clarifying the study proposal at all stages, ensuring absolute confidentiality of information as well as participant privacy and anonymity.

### Study design

This is research with a qualitative approach, and the article was prepared in accordance with the COnsolidated criteria for REporting Qualitative research (COREQ).

### Methodological procedures

Qualitative research works with the world of meanings, desires, aspirations, beliefs, values and attitudes, which corresponds to a deep space of relationships, processes and phenomena^(20)^. Qualitative research adopts a perspective of analyzing phenomena that recognizes cultural plurality and the relevance of subjects, including the voice of social actors^(21)^. It should be noted that this article is based on qualitative results of a multicenter mixed method research, with the participation of Brazil, Spain and Mexico, entitled “*Sofrimento moral durante a pandemia do covid-19 em enfermeiros: perspectivas da Espanha e América Latina*”.

### Study setting

The setting defined for the study was the Southeast region of Brazil, considering that, according to data from the Brazilian Ministry of Health Coronavirus Panel, this was the region with the most confirmed cases of coronavirus^(22)^.

### Data source

A total of 19 nurses who worked on the front line of COVID-19, in health services in southeastern Brazil, participated in the study. Nurses who worked, at some point during the pandemic, on the front line in the fight against COVID-19, were included. Participants were recruited intentionally, by expressing their desire to participate in the research, after participating in the first (quantitative) stage of multicenter research. Considering that collection was carried out during the pandemic period, a minimum period of activity was not determined. Professionals on vacation or away during data collection were excluded. The sample was defined through data saturation, which is related to the repetition of the data obtained, with the interruption of inclusion of new participants^(23)^.

### Data collection and organization

Data were collected between May and July 2021 through an individual interview following a semi-structured script, carried out remotely using Microsoft Teams^®^, which was recorded and transcribed. The interview script was constructed based on scientific literature^(14,16-19)^, and was validated in the research group, through collective construction and pilot testing. The script addressed issues related to service management and organization during the pandemic, lived experiences and ethical-moral issues and rights of patients and their family during care. The interviews lasted about 30 minutes. To record information, in order to guarantee participant anonymity, they were identified as “NUR” followed by a number according to the order in which the interviews took place.

### Data analysis

Data analysis was carried out using thematic content analysis (TCA), with the help of the ATLAS.ti software version 9. TCA allows the understanding of participants’ perception and the context in which they are inserted, through testimonials, relating semantic structures (signifiers) with sociological structures (meanings) in a process of differentiation and regrouping^(24)^.

The following chronological poles of TCA were followed: pre-analysis; material exploration; treatment, inference and interpretation of results. Pre-analysis refers to the phase of organizing the material in which skimming is carried out (first reading of the document, allowing impressions and instructions to be invaded) and exhaustive interviews to assimilate the material. Material exploration consists of coding and categorizing the *corpus*. During coding, the raw data from the previous stage were transformed into units of representation of content and its expression, which correspond, in ATLAS.ti, respectively, to codes and quotations. In categorization, the codes were grouped by their common characteristics, composing, in ATLAS.ti, what is called Family, with three categories being established, namely: Excessive working hours; Troubled relationship due to pressure from managers and the population; and Physical and mental exhaustion.

Treatment, inference and interpretation of results consists of the phase in which the analysis was deepened, establishing reflections based on the literature^(24)^.

## RESULTS

Work overload proved to be a powerful source of MD experiences related to the three categories identified as: Excessive working hours; Troubled relationship due to pressure from managers and the population; and Physical and mental exhaustion. Excess work generated and enhanced by the pandemic meant that nurses did not find an environment conducive to developing their practice as they believed to be the appropriate way, resulting in MD. As examples, nurses cited troubled dynamics of vaccination, double shift, team deficit, which creates difficulties in resting and monitoring the team of nursing technicians, the constant pressure for production targets by managers and the population itself and the impact of feelings of tiredness and mental illness on patient care.

Regarding excessive working hours, NUR3 and NUR13, nurses from Primary Health Care, report the troubled daily life that the nursing team experienced due to the work overload related to the vaccination against COVID-19 and the double shift, what prevented them from carrying out the practice as they would like.

*Health workers no longer have working hours, so you have to spend nights and weekends calling people to come, and it’s like that all the time! You see that the nursing team is extremely overworked. You have to enter data, generate data, vaccinate, provide information, assist, and most cities don’t have a team just for vaccines. The same team providing care is the same team vaccinating. So, the vaccine, everything about the pandemic right now, this stage is a mess!* (NUR3)

*He works here* [Primary Care], *he works there at the hospital. Most have double shifts. Professional relationships are frayed, and this impacts work. In the vaccine itself, I have two who work overtime, and I have another who doesn’t, and sorry for them, they only work so many hours and so on. And this all reflects on patients.* [...] *for us, from the vaccine, just COVID, we have 3 different information systems! It’s a lot of accountability!* (NUR13)

Another aspect is related to the deficit in teams, which makes it difficult to manage and monitor the team. NUR17 highlights that he was unable to take the rest period as he needed due to the lack of sufficient staff, and NUR19 reports that he was unable to perform the role of care supervisor and monitor the team of nursing technicians as he considered correct.

*There have been times when I didn’t sleep, when I spent 24 hours trying to help in other sectors, because it’s a small hospital. We only have 70 beds. So, they are divided into several CCUs and have an infirmary. I always tried to do my best and help my team who, also, due to quantity and scale, were always overloaded! We were always very overwhelmed!* (NUR17)

*This issue was difficult for us to monitor, because as the checking was manual and there were 10 beds* [...] *I ended up with 17 ICU beds, with critically ill patients, for just one nurse. So, it was very difficult! There were technicians who didn’t know how to vacuum, and we had to teach everything and, sometimes, we delegated it to another technician to do what we should do, which is to monitor, really teach, see if people are ready to undergo that procedure. So, everything we would like to do, that we would do outside of the pandemic, which is this monitoring, this supervision, we were unable to do.* (NUR19)

Regarding the troubled relationship due to pressure from managers, NUR5 exposes the management pressure for productivity, which required meeting service targets that exceeded capacity, regardless of anything.

*I don’t know about the Department* [of Health]. *As there was a health manager, and this manager is appointed by the mayor, sometimes the Secretariat puts pressure on him and he puts pressure on us. But he just wanted us to answer, answer, answer and it was number! So, there were days when there were 50 people to welcome. I don’t think that counts for that moment! People sometimes went there for nothing, as they were supposed to stay at home because of social isolation, for N reasons, but no! They went and we had to respond, because the Department of Health or the manager said that we should respond, regardless of anything.* (NUR5)

Regarding the troubled relationship with the population, NUR10 reports that the work overload generated by the population hindered carrying out the regulated work process. The participant reported that there were situations in which individuals wanted to undergo a COVID-19 test just to circumvent social isolation, and not out of protection and collective concern about transmission, and this overloaded the nurse.

*Because we have a work process. We have to do reception and notification form. If you have time* [to take the COVID-19 test], *take the test and then see a doctor. And sometimes,* [patients] *just wanted to take the test, find out the results and leave, without going through the notification form, without seeing a doctor. You continue to have symptoms and they say, “I don’t want any medication, for me, the negative is fine”. Did you understand?! I also always say, we create phrases during the pandemic, that the person just wants to know the result, they are not worried about relieving those symptoms as they continue to have them. Why? Because you want to enjoy the weekend, you want to go to a hidden party, because you don’t want to wear a mask* [...] *in short, it’s a very exhausting situation! Unfortunately! At first, this revolts! We have a moral and ethical construction, each one has their own. And when this issue, this feeling, is hurt, we feel revolted! Personally, I actually feel like I’m taking part in this dirty game they were proposing! But the other side of me says, “No! I’m not like that! I was coerced, I was pressured, anyway* [...]*”.* (NUR10)

Furthermore, NUR8 reinforces the troubled relationship with the population by mentioning having suffered threats due to the difficulties in meeting the population’s wishes.

*I think I’ve noticed that the population has become more aloof and aggressive towards us, professionals, you know?* [...] *I’ve seen it much more frequently, and we’ve been under threat of “If you don’t answer me, I’ll I’m going to film it and I’m going to call councilor so-and-so. And councilor so-and-so comes so I can get the service I want!” This has happened! We have been much more threatened, even though we often know that we are within our rights, within our issues* [...]. (NUR8)

Physical and mental exhaustion was cited by NUR13 and NUR2, when mentioning the physical and mental fatigue that overload generates in nurses, which has a direct impact on patient care.

*They are tired. Sometimes, we don’t believe that users comment that the employee did or didn’t do something that is routine for them. Sometimes he stopped doing it out of tiredness. He always has an excuse to make. It is often related to this physical and mental tiredness* [...] *there is a huge work overload.* (NUR13)

*COVID patients are very complex and require many hours of care. You have to pronate and depronate the entire shift, and this leaves us, physically and emotionally, exhausted.* (NUR2)

Mental illness was mentioned by NUR6, who relates the overload experienced by the unit manager as the reason for her taking time off from work, in addition to mentioning her own illness, recognizing it as the result of excess activities he had to undertake due to team absences due to COVID-19.

*We created a shift schedule. A new manager, who had experience in emergency care, arrived to take over this part. It was good because she was able to make that move. We managed to act appropriately or as expected. But then, when she returns to primary care again, this manager asks to leave out of time and, according to what was said to us, it’s just that she entered into a process of really becoming ill* [...] *of mental illness, of work overload!* [...] *when I was in this workplace, I was actually diagnosed with burnout! I even stayed away for a few days, about two or three months ago! Because, after you try everything, you see a lot of colleagues taking leave due to COVID. You try to work as much as possible! Sometimes, you work a little poorly, you do work for two, three* [...] *because there’s one from here, one from there, because, with this COVID thing, respiratory symptoms stay away, so it ends up that there’s always one or the other symptomatic and whoever is on the line has to end up doing everything and not be like, “Ah! My work ends here!”. We end up doing everything! I actually ended up getting sick!* (NUR6)

A similar situation was identified in NUR11’s report, when he stated that he felt unwell during the shift due to exhaustion, needing to leave to take care of his own health.

*I had a sudden illness in the unit, at night and the next day on duty in the unit too* [...] *a migraine, nausea, and I “was” with my partner, who had also fallen ill, and she said, “Are you feeling sick?”* [...] *I remember crying because I didn’t want to leave the unit* [...] *my manager coming to me and saying, “There are times when we have to stop and maybe this is your moment. You will come home!”.* (NUR11)

## DISCUSSION

The results highlighted several potential situations causing MD among nurses related to work overload during the COVID-19 pandemic. Nurses played an important role in daily work, leading the organization of strategies for the operationalization of the vaccination campaign against COVID-19^(25)^. However, participants’ reports highlighted the troubled daily life that the nursing team experienced when vaccinating the population due to work overload, which often prevented the professionals from conducting the process as they believed and is recommended in standards, which led to experiences of MD. In this regard, a study that assessed the perception of Primary Health Care nurses regarding the COVID-19 vaccination campaign development showed that the work environment was greatly impacted due to the sizing of the team responsible for vaccination, increased working hours and shortage and/or neglect of safety and health care measures, which contributed to the increase in the rate of physical and mental illness among professionals^(26)^.

Evidence points to the need for articulation and development of a strategy by actors in the health system, aiming to provide and guarantee the administration of two doses as well as adequate storage of immunobiological agents^(27)^. These new demands imposed by the pandemic converge with what was exposed by the participants of this study, who associate the greater number of activities undertaken during the pandemic with a consequent physical exhaustion, highlighting that nursing plays a fundamental role in the vaccination campaign coordination and implementation as well as in epidemiological surveillance^(28)^. In the context studied, such articulation was not possible, leaving nurses unable to act and decide according to their judgment.

Through interview analysis, it was possible to perceive that the work overload that nurses were exposed to during the pandemic impacted their professional practice and generated experiences of MD. As evidenced in the testimonies, the feeling of moral duty to care for and assist patients who needed assistance found tiredness, physical exhaustion and mental exhaustion as impediments, being a source of MD in these professionals. The reports corroborate the reflection carried out by a study that pointed out that the great demand for nursing teams’ activities leads to automated practice of processes, preventing nurses from exercising in the way they believe is correct^(29)^.

In addition to the increase in care during the pandemic, in search of higher remuneration, nursing professionals took on double working hours and were faced with excessive workloads as a result of the devaluation of nursing wages, which is still insufficient to support themselves and their families^(28)^. When trying to reconcile double working hours, professionals became worn out and felt tired, generating consequences for their health as well as losses for the institution and patient safety, as this affects their ability to provide quality and safe patient care^(30)^.

It was possible to notice in the interviews that the increase in demand for critical patients overloaded nurses, as, in addition to providing assistance, they needed to monitor and guide nursing technicians who, often, did not have adequate knowledge for the type of care needed. However, given the overload, nurses were unable to monitor and supervise nursing technicians as their professional practice requires in terms of legislation and responsibilities, thus causing experiences of MD. In this regard, a study carried out in Spain showed that professionals who worked in units converted into Intensive Care Units (ICU) had a higher degree of MD when compared to ICU professionals, due to excessive workload and difficulty in supervising inexperienced professionals in critical care, among others^(31)^. Professionals’ lack of knowledge and uncertainty about the best treatment to be carried out also proved to be sources of MD in a study^(32)^ carried out in a scenario where the absence of guidelines and protocols to be followed by professionals was a reality during the pandemic.

Regarding the power relations established in the service, it was observed that the demand from managers to care for patients in quantities greater than the teams’ capacity and the requirement to meet production targets impacted the exercise of professional practice. This occurred because nurses found themselves prevented from carrying out activities that meet important principles of professional practice, such as listening and welcoming, to follow orders aimed at productivity. This lack of institutional support was cited in a study that found that 77.5% of nurses who worked in COVID ICUs did not feel fully protected by hospital administration. Furthermore, when correlating the development of burnout, MD and symptoms of post-traumatic stress disorder, the authors noticed that these professionals presented higher rates than those who felt partially or fully supported^(10)^. In this regard, the need for nurses to perform consistent work stands out, in order to internalize institutional objectives and values, in association with the team’s needs, promoting positive behaviors that exceed norms and reflect on quality of care^(33)^.

Nursing professionals are considered protagonists of care, and have played an important role in preventing the spread of diseases as well as in health promotion, nursing diagnosis, treatment and rehabilitation^(2)^. In the context of COVID-19, in addition to all the intrinsic care of the profession, nurses needed to monitor and support patients and their families, even when experiencing inadequate working conditions. This reality fostered professional dissatisfaction and conflicts, as they felt disrespected and devalued, due to naturalization in the face of distress resulting from the work situation^(34-36)^. By allowing nursing practice in inappropriate situations, professionals violate the respect for themselves as an individual and professional and for the care necessary for their patients.

In view of this, it is essential that health institutions carry out programs and strategies to combat MD, taking into account the work overload of nurses, and it is important to be carried out collectively, considering the team in the process, such as through the creation of spaces for discussion, empowerment programs, self-care retreat and educational intervention^(37)^.

### Study limitations

This study has as a limitation the impossibility of generalizing the results, due to the geographical restriction to the southeastern region of Brazil. Although the choice was made due to the expression of confirmed numbers in the region, the diversity of the country’s regions and the decentralization of dealings regarding the pandemic, it is important to develop studies on this topic in other regions of the country.

### Contributions to nursing

The research contributes to fostering discussions and reflections on the work environment in critical contexts such as the pandemic, allowing discussions on health promotion, professionals’ quality of life and working conditions to which nurses are being subjected. In other words, it creates means for critical reflection by nurses in order to act in line with their ethical-moral values and judgments, deliberating in favor of professional practice. Furthermore, this study raises discussion about the visibility of nursing and promotes ethical awareness about understanding the work process that nurses experience and the importance of seeking to rethink environments conducive to comprehensive, humanized, ethical and responsible care.

## FINAL CONSIDERATIONS

Nurses’ work overload during the COVID-19 pandemic was identified as a potent cause of MD. Situations such as excessive and double shifts, pressure from managers and the population due to incoherent practices and physical and mental illness are obstacles for nurses to act in a manner consistent with their ethical-moral principles.

In this sense, it is necessary for health institutions to reassess the work environment organization and provide organizational strategies and policies to combat MD with a focus on nurses’ work overload, providing support and assistance to professionals. Furthermore, the need for visibility and understanding is demonstrated that the lack of adequate conditions for nursing practice generates direct consequences for care quality.
